# Supplementary material for: Propensity to childhood anxiety and depression due to exposure to adversity: A multidimensional construct
Source: PLoS One. 2025 Jun 16;20(6):e0326239. doi: 10.1371/journal.pone.0326239 (PMC12169572; doi:10.1371/journal.pone.0326239)
Supplement: Attachment — Submitted filename: Response to Reviewers.docx [file pone.0326239.s010.docx]

PONE-D-24-60103R1

Propensity to Childhood Anxiety and Depression Due to Exposure to Adversity: A Multidimensional Construct

Ronalyn M. Ramos

Dear Editor,

Thank you for your attention and time in dealing with our manuscript.

The issue raised has been addressed as per the comments sent.

Kind regards,

The authors

**##################Journal Requirements:##################**

We've checked your submission and before we can proceed, we need you to address the following issues:

1. We note that you state the following regarding your Figures: "Answer: The maps were generated using the website https://www.mapchart.net, and the final assembly was completed in PowerPoint."

Please note that MapChart states the following regarding their copyright license: "Created maps are licensed under a Creative Commons Attribution-ShareAlike 4.0 International License."

Please note that this license is more restrictive than the CC BY 4.0 license that PLOS publishes under. The CC BY 4.0 license (https://creativecommons.org/licenses/by/4.0/) means that all material on our website is freely available online, and any third party is permitted to access, download, copy, distribute, and use these materials in any way, even commercially, with proper attribution.

***Answer:*** *The specific consent from the copyright holder to publish images was required. The PLOS ONE Request for Permission form was uploaded as an other file.*

*In the figure caption of the copyrighted figure, was included the following text: “Republished from MapChart (2025) under a CC BY license, with permission from Minas Giannekas, original copyright Copyright © 2025 MapChart.”*
